# Indications for Blood Cultures in Dogs and Associations With Positive Results in 323 Submissions

**DOI:** 10.1111/jvim.70228

**Published:** 2025-08-30

**Authors:** Steven E. Epstein, Barbara A. Byrne, Jane E. Sykes

**Affiliations:** ^1^ Department of Veterinary Surgical and Radiological Sciences School of Veterinary Medicine, University of California‐Davis Davis California USA; ^2^ Department of Veterinary Pathology, Microbiology and Immunology School of Veterinary Medicine, University of California‐Davis Davis California USA; ^3^ Department of Veterinary Medicine and Epidemiology School of Veterinary Medicine, University of California‐Davis Davis California USA

**Keywords:** bloodstream infection, cardiovascular infection, endocarditis, sepsis bacteremia

## Abstract

**Background:**

Bacteremia has been associated with many diseases in dogs, but contemporary data from a large and diverse population are lacking.

**Hypothesis/Objectives:**

Report reasons for blood culture, protocols used, and diseases associated with a positive result in a tertiary referral institution. We hypothesized that larger volumes of blood, lack of previous antimicrobial administration, and changes in hematologic variables would be associated with increased rates of microbiological growth.

**Animals:**

A total of 279 dogs from which 323 blood culture results were available.

**Methods:**

For 180 submissions, patient and blood culture protocol data at the time of specimen collection were collected prospectively using a survey form. For 143 submissions, data were retrospectively collected.

**Results:**

Microbial growth was noted in 55/323 (17%) submissions, with 35/55 (63.6%) interpreted as clinically relevant growth and 20 (36.4%) interpreted as contamination, resulting in an overall positive rate of 10.8%. Specimen volume (*p* = 0.01), white blood cell count (*p* < 0.001), and neutrophil count (*p* = 0.001) were positively associated with relevant growth. Diseases associated with relevant growth were suspected discospondylitis (30%, *p* = 0.05) and illness while on immunosuppressive drugs (44%, *p* = 0.004). Submissions performed to assess for bacteremia as a secondary cause of immune‐mediated disease were less likely to yield relevant growth (0%, *p* = 0.004) than those performed for other reasons.

**Conclusions and Clinical Importance:**

In this population, blood cultures were most likely to provide diagnostically useful information in dogs with suspected discospondylitis and those receiving immunosuppressive drugs. Specimen volume should be maximized to increase the likelihood of clinically relevant growth.

## 1 Introduction

Bacteremia has been reported in normal dogs [1], as well as in dogs with a variety of disease conditions including neutropenia associated with chemotherapy [2], infective endocarditis [3], discospondylitis and spinal empyema [4, 5], acute hemorrhagic diarrhea syndrome [6], and sepsis [7, 8]. One study of 100 critically ill dogs and cats evaluated at Colorado State University between 1985 and 1987 found that positive blood cultures were most likely to be associated with diabetes mellitus and gastroenteritis [9]. In a 2022 retrospective study of 45 dogs that had blood cultures performed at Murdoch University in Australia, no association between final diagnosis and positive blood culture result was found, but a higher proportion of immunosuppressed dogs was found in the culture‐positive group [10]. A multi‐institutional retrospective study of 701 dogs that had blood cultures performed also failed to find an association with disease state, prior antimicrobial use, or immunosuppressive treatment [11]. Fever, white blood cell count, and increased serum alkaline phosphatase activity also have been associated with bacteremia in dogs [12]. Given the emergence of antimicrobial‐resistant bacteria and more widespread use of potent immunosuppressive drugs, more contemporary studies of blood culture results from a large and diverse population of dogs are needed.

The diagnostic yield of blood culture in dogs and cats varies depending on the population studied, with positive results reported in as few as 3% of dogs with fever [13] and up to 49% of critically ill dogs and cats [9]. In two studies of blood culture results from over 500 sick dogs, positive results were found in 134/581 (23%) submissions [10] and 165/663 (25%) submissions [14]. In a recent multi‐institutional study in dogs, positive blood culture results were found in 152/701 (22%) submissions [11].

Given the decrease in mortality associated with appropriate antimicrobial drug treatment in sepsis in people [15], early identification of bacteremia in dogs and implementation of appropriate antimicrobial treatment may have prognostic and therapeutic value for individual patients and decrease inappropriate antimicrobial use, with an associated decrease in selection for resistant bacteria.

Evidence‐based protocols for the collection and handling of blood culture samples in humans have been published [16]. One of the most important factors influencing the growth of pathogens is specimen volume. The Clinical and Laboratory Standards Institute recommends collection of 20 to 30 mL of blood per site [17]. Recommendations for timing of blood collection, number of bottles collected, and timing of sample collection in relation to antimicrobial administration are available, depending on the patient population [18]. Although protocols for dogs and cats have been suggested by some authors [19], evidence‐based guidelines are lacking, owing to a lack of information in the veterinary literature. In addition, the small size of veterinary patients can preclude the collection of blood volumes recommended for humans. One report in dogs identified an increase in the detection rate of blood‐borne pathogens by 19.5% when three samples were collected instead of one sample [20]. However, to our knowledge, protocol‐related variables such as volume of blood collected, current antimicrobial use, or site of blood collection for culture have not been evaluated in dogs.

Therefore, we sought to obtain more evidence regarding patient and protocol‐related variables influencing the outcome of blood culture in dogs. Our aim was to describe and identify reasons for performing blood culture, diseases associated with positive results, and protocols used for blood culture at one academic institution. Secondary aims were to assess collection and hematologic variables available at the time of blood culture sample collection that were associated with positive blood culture results. We hypothesized that larger volumes of blood drawn for culture, lack of previous antimicrobial administration, changes in hematologic variables (e.g., increased neutrophil count), and blood cultures obtained from a vascular access device would be associated with increased rates of microbiological growth.

## 2 Materials and Methods

Our study had both prospective and retrospective data collection arms for evaluation of blood culture submissions from dogs examined from July 1, 2015, through December 31, 2017, at the William R. Pritchard Veterinary Medical Teaching Hospital (VMTH) at the University of California, Davis. For the prospective portion, dogs were enrolled at the time of specimen submission for blood culture, which required completion of a data form (Table S1) by the requesting clinician as part of the request process. Data collected on this form were: patient signalment; body weight; rectal temperature at the time of collection of the first blood culture specimen; whether the patient was treated on an outpatient basis, or, if hospitalized, the patient's location at the time of collection (intensive care unit [ICU], emergency room, or wards); whether aerobic only or aerobic and anaerobic blood culture was requested; the number of specimens and volume of blood per specimen; any current treatment with antimicrobial drugs; and the reason for blood culture. Additional data collected were results of a CBC if a CBC had been performed on the same day or day before blood culture was performed and visit outcome (alive, died, or euthanized).

The retrospective component of the study involved a procedure code search of the medical record system for all blood culture submissions to the VMTH's microbiology laboratory during the study period. Data extracted were signalment; whether culture was aerobic only or aerobic and anaerobic; number of specimens submitted; site or sites of specimen collection; collection timing; growth in each inoculated bottle; results of a CBC if a CBC had been performed on the same day or day before blood culture was performed, and visit outcome (alive, died, or euthanized).

Specimens were collected after aseptic preparation of the skin and after donning sterile gloves. Blood culture bottles containing antimicrobial neutralizing resin (BD BACTEC Plus aerobic/F, BD BACTEC Plus anaerobic/F media, and BACTEC PEDS Plus/F, Becton Dickinson, Franklin Lakes, NJ) were inoculated, with specimen volume based on bottle type (pediatric vs. adult) and type of culture ordered (aerobic vs. aerobic and anaerobic). For specimen volumes < 3 mL, bottles for pediatric use were used. Bottles for use in adults were used if specimen volume was > 3 mL. Each submission could include up to three inoculated aerobic blood culture bottles and up to three inoculated anaerobic blood culture bottles (i.e., a single submission could consist of up to six specimens). The timing and volume of specimen collection was at the clinician's discretion, with all specimens being obtained within a 24‐h period. When antimicrobial treatment was deemed urgent (e.g., when sepsis was suspected), standard operating protocol was to collect the first two specimens 10 min apart and a third specimen 1 h later, after which antimicrobials could be administered. Alternatively, antimicrobials could be administered immediately after collection of the second specimen, with the third specimen collected just before the next scheduled dose of antimicrobial.

In the microbiology laboratory, inoculated bottles were incubated at 35°C until subculture after one, two, and five days of incubation (all bottles); subculturing for anaerobic culture occurred after two and five days of incubation. Aerobic subcultures were inoculated on 5% sheep blood agar plates (Hardy Diagnostics, Santa Maria, CA) and MacConkey agar (Hardy Diagnostics) and incubated at 35°C in the presence of 5% CO_2_. Anaerobic subcultures were inoculated onto *Brucella* blood agar (PRAS Brucella, Anaerobe Systems, Morgan Hill, CA) and incubated at 35°C under anaerobic conditions. Subcultures were incubated for 5 days before a determination of no growth was made.

Bacterial growth identification was determined using a variety of techniques, including matrix‐assisted laser desorption‐ionization mass spectrometry (MALDI‐TOF; Bruker, Billerica, MA), spot tests (e.g., catalase, indole, and oxidase tests), tubed media, and microbial identification strips (API, BioMerieux, Durham, NC). Identification of fungal growth utilized subculture to potato flake agar (Biological Media Services, University of California, Davis, CA) and gross and microscopic morphology examination.

For each dog from which culture of any specimen yielded growth, results from all specimens submitted were independently reviewed by two of the investigators (SE and BB) and an assessment was made as to whether growth was clinically relevant (hereafter defined as a positive submission) or represented contamination by skin commensals, based on previous literature [19, 20]. When the investigators disagreed, a third author (JS) served as adjudicator. A submission was considered positive (i.e., yielding clinically relevant growth) if the same microbial species was identified from at least one bottle from two separate specimen collections. If growth was reported from only one bottle, the medical record was reviewed for clinical or other laboratory findings that supported relevant growth (e.g., same isolate from culture of a different source, specimen drawn from an IV catheter, primary disease identified in the dog). Bacteria generally considered contaminants when isolated from a single bottle were *Bacillu*s spp., *Cutibacterium* (*Propionibacterium) acnes*, *Micrococcus* spp., and coagulase‐negative *Staphylococcus* spp. A submission was defined as negative if no growth occurred in any bottle or if growth was considered contamination.

### 2.1 Statistical Analysis

Data were assessed for normality using a D'Agostino & Pearson normality test with commercially available software (GraphPad Prism 7.0, La Jolla, CA). Data that were not normally distributed are reported as median and interquartile range (IQR). For data with binary outcomes, groups were compared using Fisher's exact test, and 95% confidence intervals (CI) calculated. Comparisons of continuous variables between groups were made using a Mann–Whitney *U* test. *p* values < 0.05 were considered significant.

## 3 Results

**FIGURE 1:**
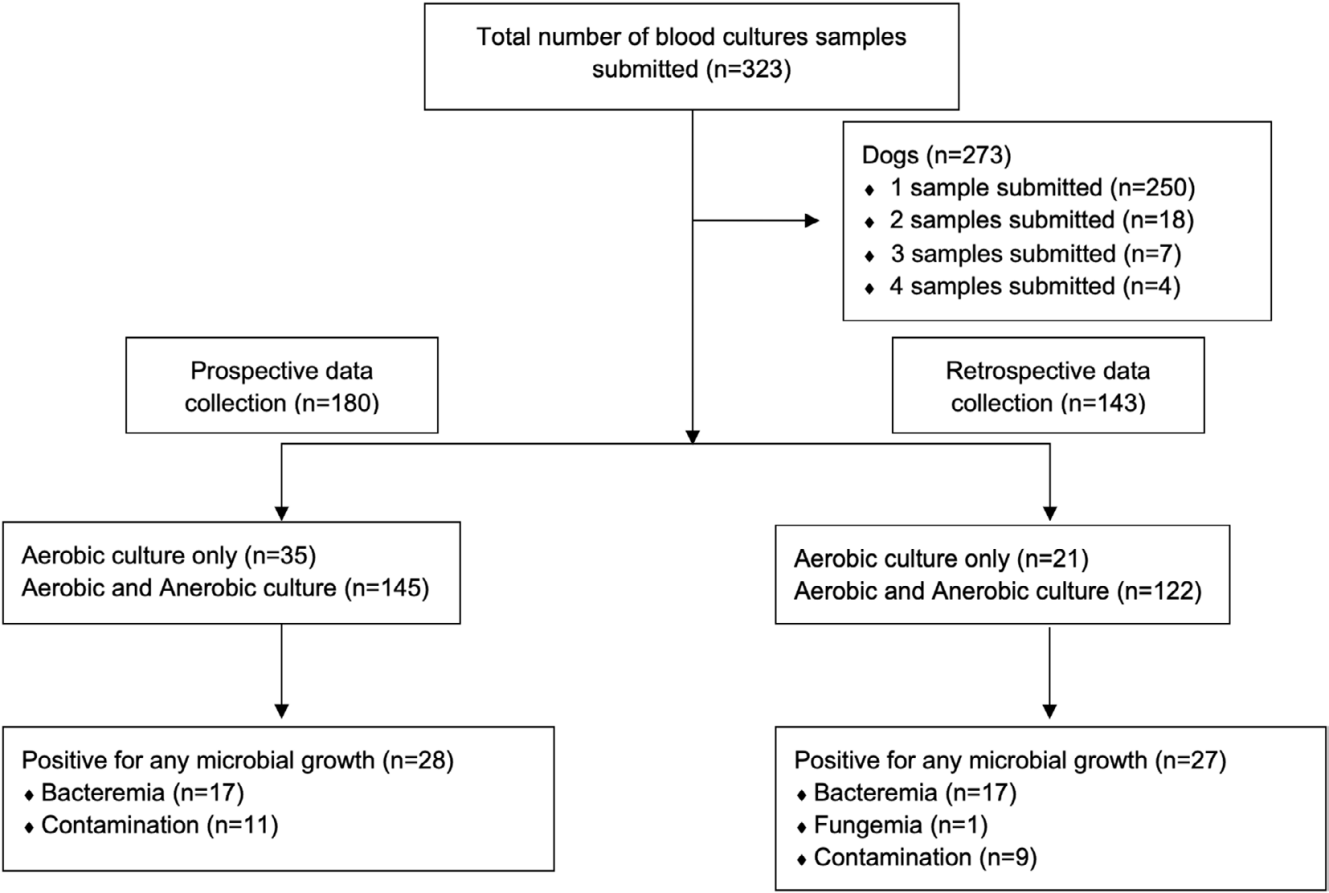
Flow diagram for number of blood culture samples, dogs, and microbial growth.

Over the 30‐month study period (July 1, 2015 through December 31, 2017), 323 blood culture submissions (each consisting of one or more specimens) were identified from 279 dogs (323 hospital visits). Four dogs had four submissions, seven had three submissions, 18 had two submissions, and 250 dogs had one submission. For 180 submissions, a completed data form was available. The remaining 143 submissions were retrieved from the medical record search (see Figure 1).

Eighty‐one different breeds were included, with mixed breed dogs (*n* = 68), Labrador retrievers (*n* = 24), German shepherds (*n* = 12), and golden retrievers (*n* = 12) being most frequently represented. One hundred thirty‐one dogs were female spayed, 106 were male castrated, 27 were intact males, and 15 were intact females. The median (IQR) age was 7 (4 to 9) years, with a median (IQR) weight of 23.9 (9.7 to 32.0 kg). Rectal body temperature at the time the first specimen was collected was available for 102 dogs, with a median (IQR) of 38.7 (38.1 to 39.6) °C.

For the dogs from which data forms were available, blood cultures were collected on an outpatient basis in 26/180 dogs; 154/180 were inpatients at the time of specimen collection. One hundred and eight of these dogs were hospitalized in the wards, and 32 were hospitalized in the ICU. Fourteen of the blood cultures were collected while the dogs were in the emergency room before admission to the hospital. For the 323 visits, 275 (85%) dogs survived whereas 48 (15%) dogs died or were euthanized during the visit in which the blood cultures were submitted.

**FIGURE 2:**
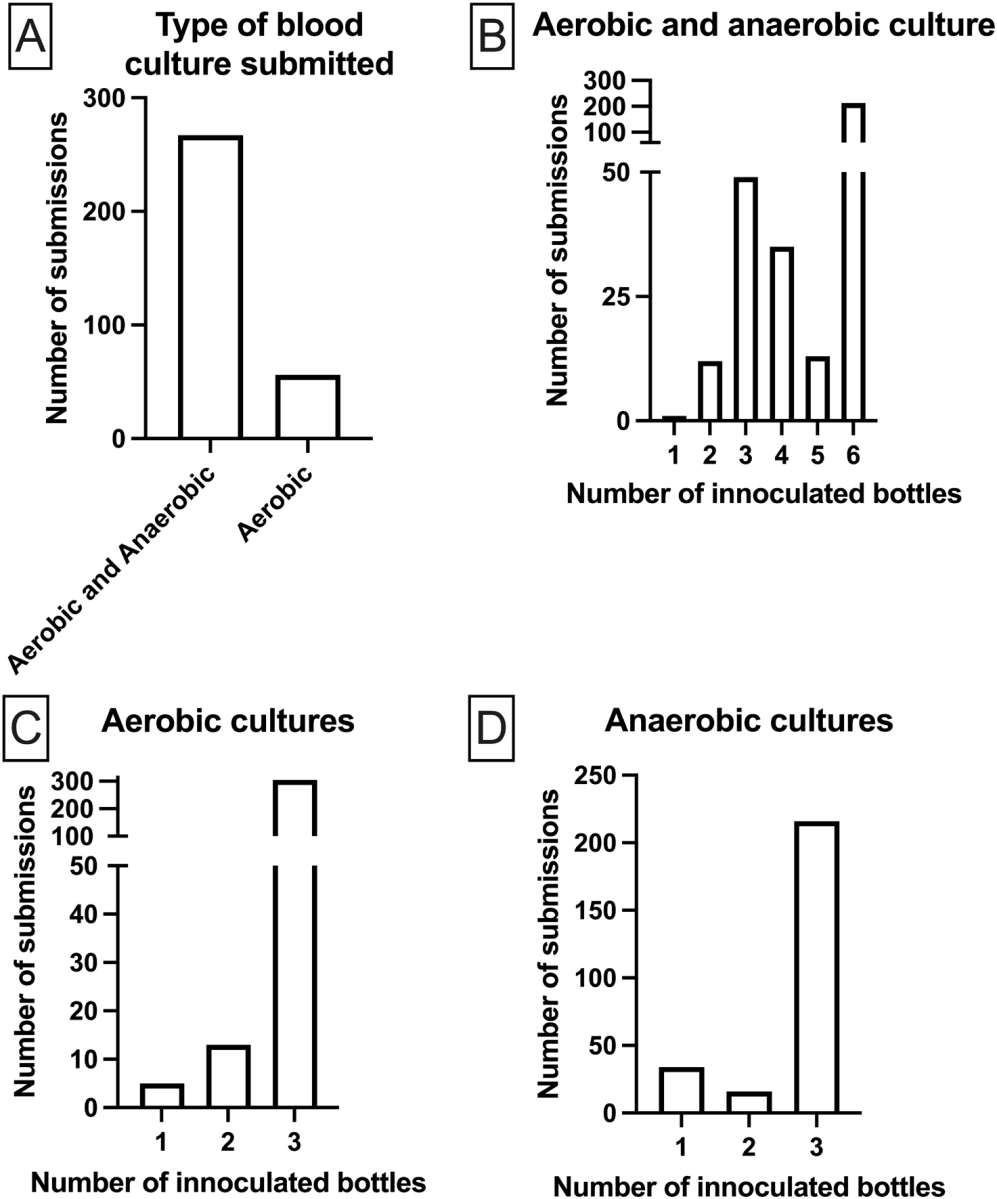
Number of submissions and associated number of inoculated bottles for aerobic and anaerobic blood culture in 323 dogs. (A) Type of blood culture submitted. (B) Number of bottles inoculated per submission for aerobic and anaerobic culture. (C) Number of bottles inoculated per submission for aerobic culture. (D) Number of bottles inoculated per submission for anaerobic culture.

Of the 323 blood culture submissions, 267 (82.7%) were for aerobic and anaerobic bacterial culture, and 56 (17.3%) were for aerobic bacterial culture only. Figure 2 shows the number of inoculated bottles per submission type.

The median (IQR) time between the first and second specimen collections was 15 (10 to 20) min with a range of 1 to 660 min (*n* = 315). The median time between the first and third specimen collections was 75 (65 to 100) min with a range of 30–960 min (*n* = 295). For seven submissions, specimens were collected from a single site. For two submissions, two different sites were used for specimen collection; for 285 submissions, three different sites were used; for six submissions, four different sites were used; and, for five submissions, site collection data were not recorded. Of the 949 collection sites identified, a peripheral vein was used 698 times, a jugular vein 186 times, and a vascular access device 65 times. The median (IQR) volume of blood inoculated into an aerobic bottle was 4.6 (3.0 to 10.0) mL (*n* = 453) and into an anaerobic bottle 5.0 (3.0 to 10.0) mL (*n* = 335). The median (IQR) total volume of blood drawn for all collections was 21 (12 to 52) mL representing 1.3 (0.9 to 1.9) mL/kg (*n* = 152).

The median (IQR) total volume of blood drawn for a submission yielding relevant growth was 31.0 (18.0 to 52.0) mL (*n* = 16). This result compared with 18.5 (12.0 to 49.5) mL for a submission that did not yield relevant growth (*n* = 136; *p* = 0.43; 95% CI of difference, −14 to 5.0). Also, no difference was found in the median (IQR) total volume of blood per kilogram body weight between submissions yielding relevant growth (1.2 [1.0 to 1.7] mL/kg; *n* = 16) and those that did not yield growth (1.3 [0.9 to 1.8] mL/kg; *n* = 136; *p* = 0.80; 95% CI of difference, −0.39 to 0.57). However, median (IQR) specimen volume per bottle was significantly higher for submissions that yielded relevant growth than for those that did not yield growth (6.0 [3.0 to 10.0] mL; *n* = 59) compared with (4.6 [3.0 to 10.0 mL]; *n* = 724; *p* = 0.01; 95% CI of difference, −2.0 to 0.0).

Treatment with both IV and PO antimicrobials at the time of specimen collection was recorded for 66 submissions and consisted of a β‐lactam, with or without β‐lactamase inhibitor (61 dogs), enrofloxacin (33 dogs), doxycycline (12 dogs), clindamycin (5 dogs), metronidazole (4 dogs), and minocycline (one dog). Thirty‐two of these 66 dogs were treated with ≥ 2 antimicrobials at the time of specimen collection.

For 268 submissions, no microbial growth was documented. Fifty‐five (17%) submissions yielded microbial growth in at least one bottle. Thirty‐five submissions yielded relevant growth (10.8% of all submissions or 63.6% of submissions that yielded any growth). Growth was considered contamination in 20/323 (6.2%) submissions (20/55 [36.4%] of submissions yielding any growth). The two investigators agreed on the relevance of growth for 54/55 (98%) submissions, whereas the third investigator made the final decision on one submission. Of the 35 submissions yielding relevant growth, 34 yielded bacteria and one yielded a fungus. Two submissions yielded polymicrobial growth and 33 yielded a single organism. The number of bottles yielding relevant growth in relation to the number of bottles inoculated is shown in Table 1. Pathogens cultured from submissions that were deemed positive were *Escherichia coli* (*n* = 8), *Staphylococcus pseudintermedius* (*n* = 7), *Staphylococcus aureus* (*n* = 5), *Staphylococcus intermedius* group (*n* = 3), *Streptococcus dysgalactiae* (*n* = 2), *Pseudomonas aeruginosa* (*n* = 1), *Campylobacter jejuni* (*n* = 1), *Streptococcus viridans* (*n* = 1), *Pasteurella dagmatis* (*n* = 1), *Klebsiella pneumoniae* (*n* = 1), *Nocardia* sp. (*n* = 1), *Bacteroides fragilis* (*n* = 1), *Enterococcus gallinarum* (*n* = 1), *Enterococcus faecalis* (*n* = 1), *Enterococcus faecium* (*n* = 1), and the fungus *Paecilomyces* sp.

**TABLE 1:** Number of blood culture bottles (aerobic and anaerobic) yielding relevant growth in relation to the number of bottles inoculated.

		Number of bottles yielding relevant growth					
		1	2	3	4	5	6
Number of bottles inoculated	1	0					
	2	0	1				
	3	2	1	2			
	4	0	0	2	3		
	5	0	0	0	0	2	
	6	5	2	3	2	0	6

The reason for blood culture was documented for 180 submissions (Table 2). The most common reasons were for identification of an underlying bacterial cause of immune‐mediated disease (*n* = 52) and for diagnostic evaluation of fever (*n* = 50). For submissions yielding relevant growth, reasons for culture were suspected sepsis [5], immunosuppressed ill dog [4], suspected discospondylitis [3], suspected endocarditis [2], diagnostic evaluation of fever [2], and a chronic wound [1]. Of the dogs that were immunosuppressed and ill, 3 were on 2 immunosuppressant medications, and 1 was on 3 immunosuppressant medications.

**TABLE 2:** Reasons for performing a blood culture in dogs and associated prevalence of positive versus negative results.

Reason for blood culture	Number with positive blood culture (%)	95% confidence interval	Number with negative blood culture	Total number of cultures
Diagnostic evaluation for immune‐mediated disease*	0 (0%)	0%–6.9%	52	52
Diagnostic evaluation of fever	2 (4.0%)	0.5%–13.7%	48	50
Sepsis with an unknown source, or source not readily accessible for culture	5 (13.5%)	4.5%–28.8%	32	37
Suspected endocarditis	2 (11.7%)	1.5%–36.4%	15	17
Suspected discospondylitis*	3 (30%)	6.7%–65.2%	7	10
On immunosuppressive treatment and new illness**	4 (44.4%)	13.7%–78.8%	5	9
Historic bacteremia	0 (0%)	0%–70.8%	3	3
Thromboembolic disease	0 (0%)	0%–70.8%	3	3
Chronic Wound	1 (50%)	1.3%–98.7%	1	2
Other^a^	0 (0%)	0%–70.8%	3	3

*Note:* Cases may appear in a category more than one time.

^a^ Includes 1 each of liver failure, large bowel diarrhea, and unknown pulmonary disease.

* *p* = 0.05.

** *p* = 0.004.

Of 18 submissions yielding growth where the location of the animal was recorded, 11 were from dogs in the wards, 4 were from dogs in the ICU, 2 were from dogs still under evaluation in the emergency room, and 1 was from an outpatient.

Five (29.4%) of 17 dogs with positive submissions had a history of antimicrobial treatment at the time of specimen collection. All dogs had been on antimicrobial treatment for no more than a single day before blood culture sample collection. Four of the dogs had a bacterial culture that was susceptible to the antimicrobial being administered, whereas one dog was being treated with only metronidazole, and *Salmonella* sp. was cultured. This result compared with 61/163 (37.4%) dogs with negative submissions (*p* = 0.60; odds ratio, 95% CI [0.70, 0.26 to 2.1]). The median (IQR) rectal temperature of dogs with positive submissions was 38.2 (38.3 to 39.3) °C (*n* = 11) which was not significantly different from dogs with a negative result, 38.9 (38.0 to 39.8) °C (*n* = 51; *p* = 0.19; 95% CI of difference, −0.4 to 2.5).

Complete blood count data were available for 252 dogs (32 with positive submissions and 223 with negative submissions) as shown in Table 3. Total white blood cell count and neutrophil count were higher in submissions with positive growth whereas band neutrophil and platelet counts were not different.

**TABLE 3:** Complete blood count data from 32 dogs with positive blood cultures and 223 dogs with negative blood cultures.

Variable	Blood culture result^a^	Median	Interquartile range	Reference interval	*p*
Total white cell count (cells/uL)	Positive	22 541	14 465–39 229	6000–13 000	
	Negative	13 760	7710–26 510		*p* < 0.001
Neutrophil count (cells/uL)	Positive	17 832	10 239–31 201	3000–10 500	
	Negative	10 582	5484–21 021		*p* = 0.001
Band Neutrophil count (cells/uL)	Positive	447	0–2032	0	
	Negative	143	0–1160		*p* = 0.19
Automated platelet count (cells/uL)	Positive	238 000	81 750–294 500	150 000–400 000	
	Negative	187 500	87 500–304 750		*p* = 0.62

^a^ Positive results indicate clinically relevant growth.

The 20/323 (6.2%) submissions that yielded growth deemed as contamination included 105 specimens (bottles). For 18 submissions, one bottle yielded growth, and for two submissions, two bottles yielded growth (each with a different microbe) for a total of 22 inoculated bottles with growth. Of the 22 inoculated bottles that yielded irrelevant growth, two had been inoculated with specimens collected from vascular access devices, and 20 had been inoculated with specimens collected by direct venipuncture. The proportion of specimens yielding contamination was 2/65 (3.1%) for vascular devices, 3/144 (2.1%) for jugular venipuncture, 7/186 (3.8%) for medial saphenous venipuncture, 7/355 (2.0%) for lateral saphenous venipuncture, 3/152 (2.0%) for cephalic venipuncture, and 0/47 (0%) for other or unspecified veins. The proportion of specimens yielding contaminants from vascular devices was not different from the proportion of specimens yielding contaminants collected by direct venipuncture (*p* = 0.66; odds ratio, 95% CI, 1.37, 0.31 to 5.4).

Of the 323 visits during which a submission was made, death was the outcome for 6 visits, euthanasia for 42 visits, and survival to discharge for 275 visits. Seven of 35 dogs with positive submissions (20.0%) did not survive, compared with 41/288 (14.2%) dogs with negative submissions (*p* = 0.45; odds ratio, 95% CI, 1.5, 0.57 to 3.6).

## 4 Discussion

In our study, we described a variety of protocols used for blood culture in dogs, as well as factors associated with blood culture outcome. Despite the availability of an institutional standard operating protocol, practices used varied considerably. Thirty‐five of 323 (10.8%) submissions yielded relevant growth, with diverse indications for collection. This result compares with rates of 14.9% to 23% in other studies in dogs [7, 10, 11, 12], although previous studies did not apply criteria to differentiate any positive growth from clinically relevant growth. In humans, the overall prevalence of blood cultures with relevant growth has been reported to be 16%–28% [21, 22, 23, 24]. As in previous studies in dogs [7, 12, 14], the most frequently isolated bacteria identified in our study were *Staphylococcus* spp. and *E. coli* .

In our study, most (82.6%) submissions included both aerobic and anaerobic cultures. In human medicine, the Infectious Diseases Society of America and the American Society for Microbiology recommend obtaining both aerobic and anaerobic cultures in adults but only aerobic cultures in pediatric patients because of limitations of blood volumes that can be safely drawn [25]. In our study, all inoculated bottles in a submission had relevant growth in 67% of submissions, with 33% of submissions having at least one inoculated bottle negative for growth. Growth of anaerobic bacteria was reported for 9% of submissions in two studies of dogs [7, 14] whereas only 1/35 (2.8%) positive submissions included an obligate anaerobe ( *Bacteroides fragilis* ) in our study. Our diagnostic laboratory stops collecting subculture samples at day 5, whereas some laboratories collect them until day 7, but it is unlikely to affect the positive growth rate of our study when comparing it to others. Requests for aerobic‐only cultures for some submissions in our study may have reflected low suspicion for anaerobic bacteremia, concerns regarding availability of a sufficient volume of blood, financial constraints, or some combination of these factors. Given the additional expense of anaerobic blood cultures and the potential sacrifice of blood volume that could be used to optimize sensitivity of aerobic cultures, rare detection of anaerobes in our study and others at our institution [3, 25] suggest anaerobic blood cultures should be reserved for situations when the history suggests an anaerobic bacterial infection (e.g., plant awn migration, aspiration pneumonia, anaerobic infection at another site). Even if these conditions exist, an antimicrobial could be selected with activity against anaerobes given their well‐documented antimicrobial resistance mechanisms and the lack of antimicrobial susceptibility testing performed.

The most common reasons for requesting blood culture at our institution were to investigate for underlying infectious causes of immune‐mediated disease, as well as for the diagnostic evaluation of fever. The most common reasons for requesting blood culture when a submission yielded relevant growth were sepsis and new illness in a dog with known immunosuppression. None of the dogs that had blood cultures performed as part of a diagnostic evaluation for immune‐mediated disease had positive blood culture results, which is consistent with a report of negative blood cultures in dogs with immune‐mediated hemolytic anemia [26]. However, because dogs with chronic bacteremia and secondary immune‐mediated hemolytic anemia have been evaluated at our institution outside of the study period, caution is advised in interpreting these findings, especially given the potentially catastrophic outcome of treatment of such dogs with immunosuppressive drugs. Current American College of Internal Medicine guidelines do not suggest blood cultures as part of the diagnostic evaluation for immune‐mediated diseases [27]. Additional studies are indicated to determine if this rationale for obtaining blood cultures should be applied to other populations.

Based on our results, blood cultures may be more likely to yield growth in clinically ill dogs receiving immunosuppressive drugs and dogs with suspected discospondylitis when compared with dogs with other illnesses. Positive blood culture results also were associated with immunosuppression in dogs in a previous study [10], and in dogs with discospondylitis in another study [28]. Therefore, if financially feasible and an infectious cause has not been identified by other means, blood culture should be prioritized for such patients; although the relevance of positive results requires further study.

We did not find an association between total volume or volume/kg body weight of blood submitted for culture and positive results. However, detection of relevant growth was significantly associated with volume of blood per bottle. In human medicine, larger specimen volumes per bottle also have been associated with increased likelihood of bacterial growth [16]. Thus, whenever possible, the volume recommended by the manufacturer should be collected and inoculated into each bottle. Regardless of patient body weight, when possible, blood culture bottles designed for adult use should be used, because these accommodate a larger volume of blood than bottles designed for pediatric use. Our results suggest that reduction in specimen volume by only a few milliliters may be enough to decrease the sensitivity of blood culture. Even in small breed dogs, this situation is unlikely to impact blood volume or hematocrit in the patient. Use of lower specimen volumes has the potential to waste client financial and hospital resources and negatively impact patient outcomes.

Dogs receiving antimicrobials at the time of blood culture were no less likely to have positive submissions than those not receiving antimicrobials. This observation is consistent with previous reports in dogs [9, 10, 11]. In both studies, however, there were few positive submissions, and this information should be interpreted with caution because a type II error is possible. As recommended for humans [25], specimens ideally should be collected before antimicrobial administration in dogs. Four of the five dogs on antimicrobials had bacteria cultured that were susceptible to the agents administered, but the antimicrobial was administered starting only one calendar day before. In our study, the blood culture system utilized bottles with antimicrobial neutralizing resin for all samples regardless of previous antimicrobial use. These resins may decrease the rate of false negative blood cultures in samples drawn from patients currently receiving antimicrobials. Our results indicate that a history of antimicrobial administration should not be a reason to avoid blood culture in dogs, but we did not evaluate long‐term antimicrobial administration.

Leukocytosis was common in dogs with positive blood culture results and consisted of neutrophilia with or without a left shift. The median white blood cell count and neutrophil count were significantly higher in dogs with positive submissions than in dogs with negative submissions, as identified in previous studies in humans [29] and dogs [9]. However, given the overlap in neutrophil counts between dogs with positive and negative blood culture results, white cell count alone cannot be used to predict the likelihood of a positive blood culture in dogs with suspected bacteremia.

We identified contamination in 6.2% of submissions, which compares with 7% in large studies in humans [30, 31]. Our microbiology laboratory provides data on all growth in the report and indicates if the microbial growth is believed to represent contamination, which is not true for all reference laboratories. This factor should be considered when comparing our results to other studies. We were unable to identify risk factors for the growth of contaminants, such as obtaining specimens from indwelling vascular catheters. In human medicine, obtaining blood cultures from indwelling vascular catheters is recommended only when a catheter‐related bloodstream infection is suspected [15]. In our hospital, vascular access devices typically are used to collect specimens for blood culture only if they are placed immediately before specimen collection and have not been used for fluid or medication administration.

The mortality rate for dogs with positive blood cultures in our study was 20% compared with 33% in a previous report of 140 dogs [7] and 1 of 9 dogs in another study [10]. We found no association between positive blood culture results and mortality in our population of dogs, in contrast to findings from large studies of humans [32, 33]. In another previous study of dogs, positive blood culture results were only associated with mortality when severe disease was present [9]. In the future, inclusion of an illness severity score may be helpful for determining an association between positive blood cultures and mortality.

Strengths of our study were the inclusion of a relatively large set of specimens for blood culture, a population with a diverse range of diseases, and the availability of detailed information regarding protocols used for specimen collection. However, because our study was performed at a single academic tertiary referral institution, our population likely differs from that in primary emergency clinics or even secondary referral hospitals Thus, it may not be possible to extrapolate our findings to other patient populations. Another limitation of our study was incomplete entries on some data collection forms. This factor contributed to a decrease in the number of data points and, in turn, the power to detect small differences in some of the variables evaluated. In addition, we relied on the results of medical record documentation, which was dependent on the attending clinician. In general, however, the medical records contained a large amount of detail. The availability of a protocol for the timing of collection and general recommendations for the volume of blood collection likely contributed to a more consistent use of the protocols than if an institutional protocol was not available.

In conclusion, the likelihood of a blood culture yielding clinically relevant bacteremia in dogs with suspected bacteremia in the population evaluated at our institution was approximately 1 in 10. Dogs suspected to have discospondylitis or those receiving immunosuppressive medications had a significantly higher likelihood of bacteremia than those with other conditions. Thus, aerobic blood culture should be prioritized for such patients. Anaerobic blood culture rarely yielded positive results, and because the likelihood of relevant growth increased with the volume of blood per culture bottle, specimen volume should be maximized for aerobic culture. Additional prospective studies are required to determine the optimal volume and timing of specimen collection for blood culture in dogs.

## Disclosure

Authors declare no off‐label use of antimicrobials.

## Ethics Statement

Authors declare no institutional animal care and use committee or other approval was needed. Authors declare human ethics approval was not needed.

## Conflicts of Interest

The authors declare no conflicts of interest.

## Supporting information


**Table S1:** Paper Data collection form that was utilized for the study.
