# Supplementary material for: Indications for Blood Cultures in Dogs and Associations With Positive Results in 323 Submissions
Source: J Vet Intern Med. 2025 Aug 30;39(5):e70228. doi: 10.1111/jvim.70228 (PMC12398271; doi:10.1111/jvim.70228)
Supplement: Supplementary file 1 — Table S1: Paper Data collection form that was utilized for the study. [file JVIM-39-e70228-s001.docx]

Supplemental Table 1: Paper Data collection form that was utilized for the study.

**Blood Culture Data Collection Form**

Enrollment: Any dog or cat that has blood cultures ordered by the primary clinician.

Primary Disease (**diagnosed or suspected**) (**Circle one**) for which blood cultures are being ordered:

Discospondylitis ☐

Endocarditis ☐

Sepsis with unknown primary source ☐

Sepsis with source that cannot be cultured ☐

List suspected source _____________________________________________

Fever of unknown origin ☐

Catheter related bloodstream infection ☐

Prior to starting immunosuppressive therapy ☐

Other ☐ ___________________________­­­­­­­­­­­­_______________________________

Is the patient currently on antimicrobials prior to the drawing of blood cultures?

Yes ☐ No ☐

If yes which one(s)?Please list dose, frequency and the duration of use

_______________________________________________________________________________

_______________________________________________________________________________

______________________________________________________________________________

Were antimicrobials given between samples being drawn for blood cultures?

Yes ☐ No ☐ If yes, what time? _______________________________________

What volume of blood was actually placed in the blood culture bottles?

Aerobic 1) ________________ml, 2) ________________ml, 3) ________________ml

4) ________________ml, 5) ________________ml

Anaerobic 1) ________________ml, 2) ________________ml, 3) ________________ml

4) ________________ml, 5) ________________ml

Anaerobic culture not performed ☐

Is this patient an:

Emergency Room patient ☐

ICU hospitalized patient ☐

ICW hospitalized patient ☐

General ward hospitalized patient ☐

Outpatient ☐

Body Temperature at start of blood culture collection __________________^o^F
